# Prevalence and risk factors of *Mycobacterium* tuberculosis complex infection in slaughtered cattle at Jos South Abattoir, Plateau State, Nigeria

**DOI:** 10.11694/pamj.supp.2014.18.1.3841

**Published:** 2014-07-21

**Authors:** Lilian Akudo Okeke, Simeon Cadmus, Ikenna Osemeka Okeke, Maryam Muhammad, Oluchi Awoloh, David Dairo, Endie Ndadilnasiya Waziri, Adebola Olayinka, Patrick Mboyo Nguku, Olufunmilayo Fawole

**Affiliations:** 1 Nigeria Field Epidemiology and Laboratory Training Programme, Abuja, Nigeria; 2 Faculty of Veterinary Public Health, University of Ibadan, Ibadan, Nigeria; 3 Federal College of Veterinary and Medical Laboratory Technology, NVRI, Vom, Plateau State; 4 National Veterinary Research Institute, Vom, Plateau State, Nigeria; 5 Department of Epidemiology and Medical Statistics, University College Hospital, Ibadan, Nigeria

**Keywords:** Risk factors, tuberculosis, cattle, abattoir, Plateau State, Nigeria

## Abstract

**Introduction:**

Bovine tuberculosis (BTB) is widespread yet poorly controlled in Nigeria hence posing a public health threat. This study determined the prevalence of Mycobacterium tuberculosis complex (MTC) and factors associated with MTC among slaughtered cattle at Jos South Abattoir in Plateau State, Nigeria.

**Methods:**

We conducted a cross sectional study in which we collected 168 lung samples systematically from 485 slaughtered cattle from May-June, 2012, and tested for acid fact bacilli (AFB) using Ziehl-Neelsen test and a duplex polymerase chain reaction technique (PCR) for MTC detection. Data on cattle socio-demographic characteristics and risk factors for zoonotic BTB infection was obtained and analyzed using Epi info version 3.5.3 to determine frequency, proportions, and prevalence odds ratios. Multiple logistic regression was done at 95% Confidence Interval (CI).

**Results:**

The mean age of the cattle was 5.6 ± 1.3 years and (108) 64.3% were females. Majority were indigenous White Fulani breed of cattle (58.5%) and about half (54.8%) were slightly emaciated. Prevalence of MTB complex was 21.4% by AFB test and 16.7% by duplex PCR. Of 33 (19.6%) lungs with lesions, 27 (81.8%) were positive for AFB; while of 135 (80.4%) lungs without lesions, 9 (6.7%) were positive for AFB. Lungs with lesions were 52 times more likely to test positive to AFB test compared to tissues without lesions (AOR=52.3; 95% CI: 16.4-191.8)

**Conclusion:**

The presence of MTC in cattle signifies its potential risk to public health. Presence of lesions on lungs is a reliable indicator of MTC infection that meat inspectors should look out for.

## Introduction

Bovine tuberculosis (BTB) is an infectious disease caused by Mycobacterium bovis a member of the Mycobacterium tuberculosis complex (MTC), which includes the closely related M. tuberculosis, the major causative agent of human tuberculosis [1]. Worldwide, tuberculosis causes about 9 million new cases and 2 million deaths annually with the sub-Saharan Africa having the highest annual risk of infection, probably as a result of the upsurge of HIV/AIDS pandemic [2]. Globally, M. bovis accounts for 10-15% of all human TB cases [2]. Bovine tuberculosis is still widespread in Africa, parts of Asia and some Middle Eastern countries [3]. In Africa, the occurrence of bovine tuberculosis due to M. bovis in humans is difficult to determine accurately because of technical problems in isolating the microorganism [4]. In West and Central Africa, where bovine TB is prevalent in animals, human TB cases due to M. bovis occur [5]. These are as a result of ingesting contaminated unpasteurized milk and raw meat and also by inhaling cough spray from infected livestock [6]. Nigeria is the most populous country in Africa and it has over 160 million people, with 19.5 million cattle and an unknown population of wildlife ruminants [7]. These factors provide an opportunity for the easy transmission and spread of bovine type of TB [7]. Nigeria has the tenth highest burden of human tuberculosis among the world's 22 countries with high Tb burden [8]. Among African countries, Nigeria has the highest estimated number of new cases with nearly 368,000 new cases annually [9]. The presence of bovine tuberculosis in Nigeria can be traced back to 1932. Several reports have pointed to the endemic nature of bovine tuberculosis in different parts of Nigeria [10–14]. Shitaye et al. [5] reported a prevalence of 8.2% in a study carried out in an eastern abattoir of the country. Although other workers [12] had presented a prevalence of 0.49% between 1989 and 1993 in a northern abattoir in Kaduna, this report was based on abattoir records. Estimates based on abattoir records without actual visits and ante-mortem or post-mortem examinations, under reports the true incidence of the disease [13]. Cadmus et al. [13] confirmed the prevalence of 8.8% in an Abattoir in the South West. Recent results reported by [13] on tuberculin skin test in selected herds in some of the states of Nigeria revealed that the prevalence ranged between 0 to 15.1%. Worldwide, there is increasing contact between humans and animals due to increasing population density and growth especially in poor developing countries where livestock offers important socioeconomic, cultural, and religious pathways out of poverty [8]. However, in developing countries like Nigeria, bovine tuberculosis is considered a neglected and poverty related zoonosis [7]. Bovine TB in humans is becoming increasingly important in countries like Nigeria as humans and animals share the same micro-environment and dwelling premises especially in rural communities [15–17]. The disease has a major economic impact on livestock productivity, and also contributes to pulmonary tuberculosis in humans: 10 to 15% of human tuberculosis is considered to be caused by bovine tuberculosis [7]. In view of the great economic impact of bovine TB in animals and the potential risk of transmission to human especially people living with HIV, it is necessary to determine the prevalence of BTB and assess the risk factors for its transmission. The results of this study will increase the available empirical data on bovine tuberculosis in Nigeria and provide baseline information for the development of guidelines and interventions aimed at controlling the transmission of bovine tuberculosis.

## Methods

**Study Area:** The study was conducted in Plateau State, Nigeria. The state is made up of three agricultural zones and seventeen Local Government Areas. The state is located in the North Central geopolitical zone of Nigeria. It is located on latitude 8.50 and 10.46 East and longitude 8.20 and 10.36 West with a total land area of 26,899 square kilometres. The climate of the state is tropical with a cold period between November and February. The vegetation is Guinea savannah and is conducive for livestock, poultry and crop production. Indigenes of Plateau State are mainly engaged in agriculture. The major livestock produced in the state include; cattle (including exotic breeds), sheep, goats and pigs. Majority of the cattle in the state is owned by the Fulani pastoralists, who are widely spread across the state.

**Study design:** A descriptive cross sectional study design was employed to determine the prevalence and assess the risk factors of Mycobacterium tuberculosis complex among slaughtered cattle at the Jos South abattoir.

**Study population:** The study population were cattle slaughtered at the Jos South Abattoir in Plateau State.

**Sample Size:** Aliyu MM et al. [9] found a prevalence rate of 12.5% in Gombe, Nigeria which was used to calculate the sample size of 168 for cattle.

**Sampling technique:** Cattle were selected using a systematic sampling technique. We visited the abattoir twice a week having a pre-information that approximately 40 to 45 cattle were slaughtered daily. At each visit, lung tissues were taken from every other cattle slaughtered, making a total of 20 out of 45 cattle slaughtered daily.

**Data collection tools:** A structured self administered questionnaire was used to obtain information on demographic characteristics of cattle such as the breed, sex, and age (years), body conformation of cattle: categorized into: Apparently healthy (AH); Slightly emaciated (SE) or Emaciated (E) depending on visual assessment of their body information and lesions from tissue were scored as 1+, if only one organ was affected and the nodules were miliary that is, still embedded; 2+, if only one organ, but with pronounced nodular lesions; 3+, if more than one organ was affected.

**Sample collection:** Lung tissue biopsies with or without visible lesions were collected from each cattle. Tissue biopsies were collected using sterile scissors and forceps and deposited into a clean, sterile and labelled wide mouthed container with top screw caps. The samples were transported to the laboratory on ice packs for analysis. Samples that could not be processed immediately were kept at -800C in Bacterial Research Laboratory prior to processing.

**Data analysis:** Data was entered and analyzed using Epi info 3.5.3 version software and Microsoft Excel to determine frequency, proportions, prevalence ratio, prevalence odds ratio and Chi Square at 95% confidence interval was used to assess risk factors for Mycobacterium tuberculosis complex infection.

**Laboratory method:** Samples were processed at the Federal College of Veterinary and Medical Technology (Histology laboratory), NVRI, Vom according to [18].

**Sample processing for tissue biopsies:** Tissue biopsies were used directly to make a smear on a clean grease free micro slide using a sterile wire loop.

**Procedure:** Smears were heat fixed and allowed to cool. Slides were flooded with strong carbol fuschin and heated gently avoiding boiling and allowed for cool. Slides were rinsed with water Slides were flooded with 3% acid alcohol for 5 minutes. Slides were rinsed with water. Slides were flooded with 0.5% Malachite green for1 minute. Slides were rinsed with water and thereafter air dried. Oil immersion was applied and slides were viewed microscopically using ×100 objective.

### Polymerase Chain Reaction Technique

#### DNA extraction from tuberculosis and non tuberculosis lung tissue

**Tissue homogenization:** Using sterile forceps and blades, 2 gm each of all the tissues collected were transferred into sterile pestle and mortar containing sterile sand and ground together.1.5mls of phosphate buffer saline (PBS) was added to the ground tissue and mixed well to form a suspension. The suspension was transferred without sieving into 1.5ml clean properly labelled micro centrifuge tubes. These were stored at -20°C until needed for DNA extraction.

**DNA Extraction from Tissues:** DNA extraction was carried out using kit extraction (Zymo Research^®^ country South Africa) according to manufacturer's instruction. Samples stored at -20°C were removed and allowed to thaw at room temperature. Tissue homogenates were centrifuged at 10,000rpm for 5 minutes to obtain clear supernatant. Supernatant was transferred into a clean micro centrifuge tube. 10µl ZymoBeadsTM was added to the supernatant, mixed by inversion and incubated at room temperature for 5 minutes. Mixture was centrifuged at 1,500rpm for 1 minute. Supernatant was discarded. 200µl of genomic lysis buffer was then added to the ZymoBeadsTM, resuspended thoroughly by pipetting up and down, centrifuged at 1,500rpm for one minute and after which the supernatant was discarded. 200µl of DNA pre-wash buffer was added to the ZymoBeadsTMand the pellet resuspended, transferred into a new tube and centrifuged at 1,500rpm for 1 minute after which the supernatant was discarded. This was followed by the addition of 500µl of g-DNA wash buffer to the spin column and centrifuged at 1,500 rpm for one minute. Supernatant was discarded and pellet recentrifuged. The spin column was then transferred to a clean micro centrifuge tube and 70µl of DNA elution buffer was added and then centrifuged at 10,000rpm for 1 minute. The eluted DNA (70µl) was stored at -20°C until needed for molecular applications.

**Primer Selection for PCR:** Duplex PCR for MTB Complex and M. bovis The duplex PCR (two sets of primers) that has the capacity to detect both MTB complex organisms and M. bovis at the same time was employed. The first set comprised of forward ISN1 (5'CGTGAGGGCATCGAGGTGGC-3’) and reverse ISN2 (5’-CGTAGGCGTCGGTGACAAA-3’ primers amplifying a 260 bp genomic fragment of insertion sequence ISN specific for MTB complex. The second set contained forward JB21(5’-TCGTCCGCTGATGCAAGTGC-3’) and reverse JB22(5’-CGTCCGCTGACCTCAAGAAAG-3’) primer amplifying a 500 bp genomic fragment specific for M. bovis. The JB primers detect M. bovis, while the INS detects the MTB complex [19].

**Polymerase chain reaction (PCR):** The master mix for 1 reaction contained 16µl nuclease free water, 25µl of 2X Dream tag DNA polymerase, 1µl of template DNA, 1µl (10pmol/ml) of JB21 and JB22 (Forward and Reverse) each respectively, 1µl of INS1 and INS2 (Forward and Reverse) each respectively. Amplification was initiated by initial denaturation at 95°C for 5 minutes and was followed by 30 cycles of 96°C for 1 minute (final denaturation), 68°C for 1 minute (Annealing), and 72°C for 1 minute (Initial extension). This was followed by 72°C for 7 minutes (final extension). The PCR was carried out in Thermal cycler (Applied Biosystem-Gene Amp PCR system 2700)

**Electrophoresis:** PCR products were fractionated electrophorectically in 1.5% agarose gel in Ix TBE buffer, pH 8.3 for 1 hour 30 minutes, and visualised under UV light using an image documentation system (Syngene) after staining with ethidium bromide. The staining was done by immersing the gel in a solution of ethidium bromide in a shaker for 15 minutes. The size of the amplicon was determined by comparison with 260 bp DNA ladder.

**Ethical consideration:** Ethical clearance was obtained from the Health Research Ethics Committee in Plateau State (PSSH/ADM/ ETH,CO/2012/40) and Permission was obtained from the Head of Veterinary Services, Plateau State.

## Results

A total of 168 lung tissues with or without visible lesions were collected from 485 cattle slaughtered at Jos South Abattoir between May and June, 2012. The mean age of cattle was 5.6 years ± 1.3 years), 108 (64.3%) were females. Majority, 98 (58.3%) and 98 (58.3%) were indigenous and white Fulani cattle respectively, and 94 (54.8%) were slightly emaciated. Age group >5 (years) recorded 85 (50.6%) (Table 1).

**Table 1:** Characteristics of cattle slaughtered at Jos South abattoir, Plateau State, Nigeria, 2012

Characteristics	Frequency	Percent
**Age of cattle (years)**		
≤ 5	83	49.4
>5	85	50.6
**Breed of Cattle**		
Red Fulani	70	41.7
White Fulani	98	58.3
**Sex**		
Female	108	64.3
Male	60	35.7
**Source of Cattle**		
Indigenous	98	58.3
Northern Nigeria	70	41.7
**Body Conformation**		
Apparently healthy	52	31.0
Emaciated	24	14.3
Slightly Emaciated	94	54.8
**Presence of lesion on lung tissue**		
Yes	33	19.6
No	135	80.4
**Scores for lesion**		
+1	27	84.4
+2	5	15.6

The prevalence rate of Mycobacterium tuberculosis complex by Zeihl-Neelsen staining technique was 21.4%, while the prevalence rate of Mycobacterium tuberculosis complex was 16.7% by duplex polymerase chain reaction (Table 2). As body conformation score decreased from apparently healthy to slightly emaciated and then to emaciated, the risk of a positive AFB test increased (X2= 8.40; df=3, P < 0.05). The cattle that were slightly emaciated and emaciated are eight times (POR= 8.17; 95% CI: 1.67-44.97) and six times (POR= 6.09; 95% CI:1.62-26.94) respectively, at greater risk of a positive result to AFB test when compared to apparently healthy animals. There was no significant difference in the prevalence of bovine tuberculosis, as regards age, sex, breed and source of cattle (Table 3). Table 4 shows the unconditional logistic regression of risk factors and result of acid fast bacilli test for the cattle. The risk of a positive result increased with the presence of lesions on lung tissues (adjusted POR= 52.31; 95% CI: 16.38-191.82).

**Table 2:** Ziehl Neelsen test results and detection of MTBC from lung tissues positive for AFB test by Polymerase Chain Reaction Technique among cattle slaughtered at Jos South abattoir, Plateau State, Nigeria, 2012

Result	ZN Test Frequency	Percent (%)	PCR Frequency	Percent (%)
Positive	36	21.4*	30	83.3
Negative	132	78.6	6	16.7*

MTBC: *Mycobacterium tuberculosis* complex, AFB: Acid fast bacilli

* Prevalence rate by ZN test = 21.4%

* Prevalence rate by duplex PCR = 16.7%

**Table 3:** Association between different risk factors and result of acid fast bacilli test for cattle slaughtered at Jos South Abattoir, Plateau State, Nigeria, 2012

Characteristics	No. Positive (%)	No. Negative (%)	POR*(95%CI)
**Age group (years)**			
≤5	19 (22.9)	64 ( 77.1)	1.19(0.53-2.65)
>5	17 (20.0)	68 ( 80.0)	
**Sex**			
Female	26 (24.1)	82 (75.9)	1.59(0.66-3.86)
Male	10 (16.7)	50 (83.3)	
**Breed of Cattle**			
Red Fulani	20 (24.1)	50 (71.4)	2.05 (0.91-4.62)
White Fulani	16 ( 16.7)	82 (83.3)	
**Source of Cattle**			
Indigenous	16 (16.3)	82 ( 83.7)	0.49 (0.22-1.09)
Northern Nigeria	20 (28.6)	50 (71.4)	
**Presence of lesion on tissue**			
Yes	27 (81.8)	6 (18.2)	**63.0 (20.7-191.8)**
No	9 (6.7)	126 (93.3)	
**Body condition**			
Apparently healthy	3 (5.8)	49 (94.2)	1.00 (referent)
Slightly emaciated	8 (33.3)	16 (16.7)	**8.17 (1.67-44.97)**
Emaciated	25 (27.2)	67 (72.8)	**6.09 (1.62-26.94)**

* POR-Prevalence Odds Ratio

**Table 4:** Unconditional logistic regression of risk factors and result of acid fast bacilli test for cattle slaughtered at Jos South Abattoir, Plateau State, Nigeria, 2012

Characteristics	*APOR(95%Confidence Interval)	p value
**Presence of lesion** Yes No	52.31 (16.38-191.82)	0.0001
**Body conformation of cattle** Apparently Healthy Emaciated	0.54 (0.13-2.32)	0.41

* APOR – adjusted Prevalence Odds Ratio

*Age and Sex had been controlled for under the unconditional logistic regression model

## Discussion

This study shows that almost all the cattle slaughtered were from the nomadic herds located within Plateau State. Sometimes cattle from neighboring countries like Chad, Cameroun, and Niger are slaughtered in the abattoir. This corresponds with the report of [20]. The prevalence of Mycobacterium tuberculosis complex by acid fast bacilli test and by duplex polymerase chain reaction in this study were higher than the prevalence of 4.4% recorded by [20] in a research conducted in at Jos South abattoir and Bukuru cattle market in Plateau State, where samples were analyzed using the PCR technique known as deletion analysis. Also, the prevalence of bovine tuberculosis, in Cross-river state abattoir in Nigeria was 1.3% [21], while 2.1% was reported in Maiduguri abattoir also in Nigeria [22]. However, in Sokoto prevalence found were much lower and were 0.4, 0.5 and 0.7% [16, 12, 23]. In Ogbomoso area of Oyo State a prevalence rates was 5.0% of M. bovis based on tuberculosis testing and 1.7% based on acid fast staining [24]. In Cameroon a prevalence rate of about 6% was reported in slaughtered cattle [5] while in Chad from slaughter house studies approximately 9% of all inspected carcasses were condemned because of bovine TB [18]. A prevalence rate of 11.5% was found in a comparative intra dermal tuberculin study [25, 26] reported a prevalence of 13.2% in Tanzania using tuberculin testing. All these studies have shown that bovine tuberculosis is still endemic in many African countries. The higher prevalence obtained in this study might be because of failure to adopt the test and slaughter policy in Nigeria, and the lack of testing/ examination of importation from neighboring countries (Cameroon, Chad and Niger) as a result of lack of control of border and inadequate quarantine measure. The increase in intensive farming practice where large herds are housed together for long periods and poor hygiene may also contribute to the spread and endemicity of the disease [17]. TB prevalence may be underestimated in TB cattle because of undetected lesions in early infection, or because small lesions might be missed or because meat inspectors are pressurized by butchers to ignore [17].

Older aged cattle which were above 5 years of age and females were slaughtered more during the study period and they were the mostly affected by tuberculosis lesions. This may be due to the fact that cattle owners in the study area preferred to keep more females than male animals in their herds [21]. Anon in the Annual report of the Ministry of Agriculture and Forestry Resources (1976) [21] also reported that the female cattle stay longer in the herd for the purpose of reproduction. However, the white Fulani cattle were slaughtered more during the study period. There was no significant difference in the prevalence rate of bovine tuberculosis in cattle with respect to age, sex, breed and source of cattle which corresponds to the findings reported by [27] a work conducted in Ethiopia, but different from the findings in Republic of Ireland by [28]. The risk of a positive result increased with the presence of lesions on lungs as reported by [5]. The presence of caseous and/or calcified lesions and even lesions resembling tuberculosis lesion may not always turn out to be of mycobacterial origin. In contrast, calcified lesions can be caused by other intracellular organisms or parasites. This may be the reason for their failure to detect by the duplex polymerase chain reaction technique among the lung tissues with visible lesions. It may also have been affected by the sample taking technique during smear preparation as Mycobacteria are not evenly distributed in the tissue samples. In most cases, TB lesions in cattle in particular may not always be observed, therefore the absence of visible lesions may not always mean absence of Mycobacterium tuberculosis complex infection as isolation of M. bovis has been confirmed from non-visible lesions [25], this corresponds to the findings of 9 (6.7%) of lung tissues without visible lesions being positive for bovine tuberculosis in this study.

Most polymerase chain reaction assay amplifies fragments from M.tuberculosis complex and thus cannot distinguish between infections caused by M.tuberculosis and M. bovis. Routine application of PCR based method requires that the target sequence should be highly specific and should be present in all the strains tested [29]. PCR using primers JB21/JB22 has been considered to be highly reliable in identifying M. bovis isolates, presenting 100% concordance with the conventionally microbiological method [19]. However, the absolute specificity of JB21/ JB22 primers to M. bovis has been disputed by other study that reported that 13.3% of M. bovis isolates failed to produce the 500bp fragment [30]. Using specific primers for IS6110 sequence, the 500 bp-negatives isolates were identified as belonging to the Mycobacterium tuberculosis complex suggesting that the isolates may lack the genomic target for JB21/JB22 primers. For this, reason MTB complex which comprises of M.tuberculosis, M. bovis, M.caprae and M.africanum strains were detected by the ISN1 and ISN2 while the JB21/JB22 primers which is specific for M. bovis detection could not detect the M. bovis strain. However, Naima et al. [23] reported that the detection of TB in cattle either by Acid fast bacilli test or PCR is suggestive of M. bovis, hence further molecular work like spoligotyping and variable number of tandem repeats (VNTR) to differentiate strains are recommended

## Conclusion

The presence of MTB complex in cattle signifies its potential risk to public health. Acid fast bacilli positivity increased with the presence of lesion on tissue from slaughtered cattle. Health education of the public on bovine TB is urgently required. Also, active surveillance for MTB complex and enforcement of test and slaughter policies at abattoirs are recommended
